# Portuguese version of the Climate Change Distress and Impairment Scale

**DOI:** 10.1192/j.eurpsy.2025.2007

**Published:** 2025-08-26

**Authors:** A. T. Pereira, C. Cabaços, C. C. Marques, M. J. Soares, A. I. Araújo, A. Macedo

**Affiliations:** 1 Institute of Psychological Medicine, Faculty of Medicine University of Coimbra; 2 Institute for Biomedical Imaging and Translational Research (CIBIT), University of Coimbra, Coimbra; 3 Institute for Biomedical Imaging and Translational Research (CIBIT), University of Coimbra, Coimbraimbra, Portugal

## Abstract

**Introduction:**

The term “climate change distress” has been proposed to capture the broad range of negative emotional reactions associated with Climate Change/CC. Although it can be an adaptive reaction that motivates climate action to mitigate a real threat, for a growing number of people climate distress impairs subjective well-being and daily functioning, causing significant suffering to such an extent it often necessitates psychological support.

Hepp et al. (2023) developed the Climate Change Distress and Impairment Scale/CC-DIS, comprising 23 items to distinguish CCDistress (spanning eco-anxiety, -anger, -sadness) and CCImpairment that may indicate a need for intervention.

**Objectives:**

To analyse the psychometric properties of the Portuguese version of the CC-DIS.

**Methods:**

Participants were 590 adults, recruited through social media (64.6% women; Mean age=34.40±16.18; range: 18-75) completed an online survey including: preliminary Portuguese versions of CC-DIS; Pro-Environmental Behavior Scale/PEBS (Pereira et al. 2024); and Depression Anxiety and Stress Scales-21/DASS-21 (Pais-Ribeiro et al., 2004). Total sample was randomly divided into two sub-samples: one (*n*=290) for exploratory factor analysis/EFA and the other (*n*=300) for confirmatory factor analysis/CFA

**Results:**

**RESULTS and DISCUSSION:** EFA and parallel analysis resulted in two factors (explained variance= 58.60%). CFA of this two-factor model resulted in “acceptable” fit indices (χ2/df=2.772; CFI=.892; TLI=.872; GFI=.846; RMSEA=.0796; *p*<.001), with standardized factor loadings ranging from .490 to .844. Cronbach’s alpha coefficients were .90 for CCDistress and 0.85 for CCImpairment.

CCImpairment, but not CCDistress, correlated significantly and moderately (*r*=.25) with DASS-21, suggesting that these last two may be relatively independent phenomena.

With PEBS, the correlation coefficients were both significant (*p*<.001): .43 for CC-Distress, .22 for CC-Impairment. Both CCDistress and CCImpairment were significant predictors of collective (R^2^=17.5%; b) and political (R^2^=17.2%; b) pro-environmental actions, but only CCDistress predicted personal actions (R^2^=22.9%, *p*<.001; ), which suggests the more maladaptive nature of CCImpairment.

Such as reported by Hepp et al. (2023), CCDistress mean scores were moderate to high (3.71±0.63); CCImpairment were low to moderate (2.13±0.72) and only the former significantly differ by gender (higher in women). This suggests that individual factors, other than gender (e.g., personality traits), may be more relevant to understand psychopathological reactions to CC.

**Conclusions:**

The Portuguese CCDIS presented good validity (construct, convergent and discriminant) and reliability. It can be used to better understand and improve CC adaptation in our country, which, like Spain, is located in Southern Europe, the area of the globe projected to to be at the highest risk for CC negative impacts.

**Disclosure of Interest:**

None Declared

